# Twenty years after storm Gudrun: A lasting impact on forest science that highlights the critical role of forest monitoring

**DOI:** 10.1007/s13280-025-02159-z

**Published:** 2025-03-22

**Authors:** Luis Andrés Guillén, Adam Felton

**Affiliations:** Southern Swedish Forest Research Centre, Swedish University of Agricultural Sciences, Box 190, 234 22 Lomma, Alnarp Sweden

**Keywords:** Environmental monitoring, Forest damages, Forest disturbances, Forest sciences, Storm Gudrun, Sweden

## Abstract

Storm Gudrun represents one of the most significant meteorological events in recent Swedish history, causing widespread forest destruction in southern Sweden. Now, 20 years after the storm, it is time to reflect on its impact on the forest sciences. We highlight key research themes that emerged in response to the storm, emphasising the critical role of research and monitoring infrastructure in facilitating the generation of valuable insights. We identified 18 articles focusing on storm Gudrun within forest-related research fields, which were, in turn, cited by over 600 subsequent studies. A citing network analysis resulted in eight research clusters, including, wind dynamics, carbon dynamics due to disturbances, spruce bark beetle responses, and storm damage in Sweden and Europe. Our review of primary literature underscores their dependence on established environmental monitoring and research infrastructure to provide essential background data. Maintaining these infrastructures is central to the advancement of forest-related sciences.

**Supplementary Information:**

The online version contains supplementary material available at 10.1007/s13280-025-02159-z.

## Introduction

Storm Gudrun, which struck southern Sweden between 8 and 9 January 2005, represents one of the most significant meteorological events in recent Swedish history. Wind gusts reached velocities of up to 42 ms^−1^ (Skogsstyrelsen 2006), resulting in extensive societal disruptions, including widespread power outages, interruptions to transportation networks, and the failure of critical infrastructure affecting a population of thousands of people (Skogsstyrelsen 2006). The storm’s most severe environmental impact was the large-scale destruction of forests in the Götaland region, specifically in the counties of Halland, Smaland, and Scania, with an estimated 70 million cubic metres of timber felled—equivalent to Sweden’s total annual harvest under typical conditions, and with an economic cost assessed at up to EUR 3.2 billion (Broman et al. 2009).

While forest damage from storms is a relatively common occurrence (Patacca et al. 2023), an event of the magnitude and impact of storm Gudrun is rare. The extensive and unprecedented consequences of this storm attracted significant attention within the forestry sector (Ulmanen et al. 2015), catalysing discussions on storm-related risks and mitigation strategies (Valinger et al. 2019). Over time, storms and their associated impacts have become critical topics in forest research and education. Now, two decades later, it is timely to reflect on the legacy of storm Gudrun to forest science. This short communication aims are twofold: (1) to provide an overview of key research themes that have emerged in response to the storm and (2) to highlight the critical role that research and monitoring infrastructure has played in enabling the generation of valuable insights for forest sciences from this single destructive event.

## Materials and methods

### Literature search

We conducted a comprehensive search for scientific articles addressing storm Gudrun using the Web of Science Core Collection database. Our search consisted of two distinct but overlapping efforts that consisted of one search that focused on scientific articles in general, and one search that focused on scientific articles involving forests. We did so in order to place the forest-related studies within the larger scientific context. In August 2024, an initial general search was performed with the query: “TS = ((storm OR hurricane OR cyclone) NEAR/2 (Gudrun OR Erwin))”, yielding 34 “primary” articles (32% with an open-access designation). The term “Erwin” was included as a search term because this name was used to refer to the storm in some other European countries (e.g. Germany). Subsequently, using the Web of Science tool “citation analysis”, we identified additional articles citing the primary set of documents. The results were refined to include only “Articles” and “Review Articles”, resulting in 973 documents (54% with an open-access designation). Moreover, given our specific interest in the storm’s impact on forest-related sciences, the articles that resulted from the primary general query were manually filtered to include only articles that focused on scientific fields that work with forests. This refinement narrowed the dataset to 18 primary articles related to forests (44% with an open-access designation), which served as the basis for another round of citation analysis. Following a similar refinement process to exclude self-citations and focused on “Articles” and “Review Articles”, the final dataset comprised 647 “citing” articles (60% with an open-access designation). The results from each single search were exported with their full records and cited references for further analysis. Please refer to the Supporting Information for a complete list of the articles included in our analysis.

### Network analysis

We carried out a citation network analysis in order to understand how strong the relationships between the different articles were. We created two citation network maps utilising the open-access visualisation software VOSviewer.^1^ The programme calculates similarities between specific data in each item (in our case citation data) which is then represented graphically in a low-dimensional space (for specifics on the software and its methodology please refer to Van Eck and Waltman (2010)). The first citation network map was done using the documents gathered from the primary general search including both source and citing articles. The second citation network map was carried out using the 647 documents that focused on forests. At this point, the large amount of small clusters made the analysis difficult, due to many documents being cited only a few times and giving more noise to the map. Additionally, several documents served as “super connectors” across the clusters, being cited by many different papers in several clusters. These “super connectors” were highly cited (> 500 times) and published in journals as Nature, or on one occasion an assessment report from the IPCC. Hence, a new network map analysis was carried out excluding the papers that had been cited less than 10 times (389 papers) and the “super connector” papers (5 papers). Another four papers were not connected to any cluster and were thus omitted. The final number of papers in the forest-focused map was 380 papers, with a minimum cluster size of 25 papers.

We labelled each cluster trying to describe their main scientific topic or other notable characteristics. First, we scanned the three articles that had the most connections, the most citations, and the original article present that focused on the Gudrun storm. Secondly, we carried out a frequency word count of all the cluster articles titles, abstracts, and keywords, noting which words and combination of words provided a logical thematical category. Cluster names are hence an effort to characterise sets of articles with large variability in subfields, scope, and methods.

### Focused literature review

Finally, we read and categorised the 18 primary articles that were focused on forestry to map the article type (e.g. empirical, modelling, and mixed), methodological approach (qualitative, quantitative, or mixed), type and sources of data (e.g. empirical and pre-existing datasets), and funding agencies of the research (e.g. country, public or private, and specific governmental agencies). These classifications were used to provide insights regarding the importance of environmental monitoring efforts for carrying out the research focused on the storm Gudrun. We considered that any paper that relied upon data collected prior to the storm, could not, or would be very difficult (logistically and financially) to be recreated after the storm. We do, however, acknowledge that with enough financing and effort, one could in some cases develop proxies for data from before the storm. In other cases for which the published articles did not depend explicitly upon such “before” data, it is reasonable to assume these studies could have still been conducted, although it is possible they were still indirectly reliant on other “beforehand” data collection that we did not identify.

## Results and discussion

### Science across fields and countries

**Fig. 1:**
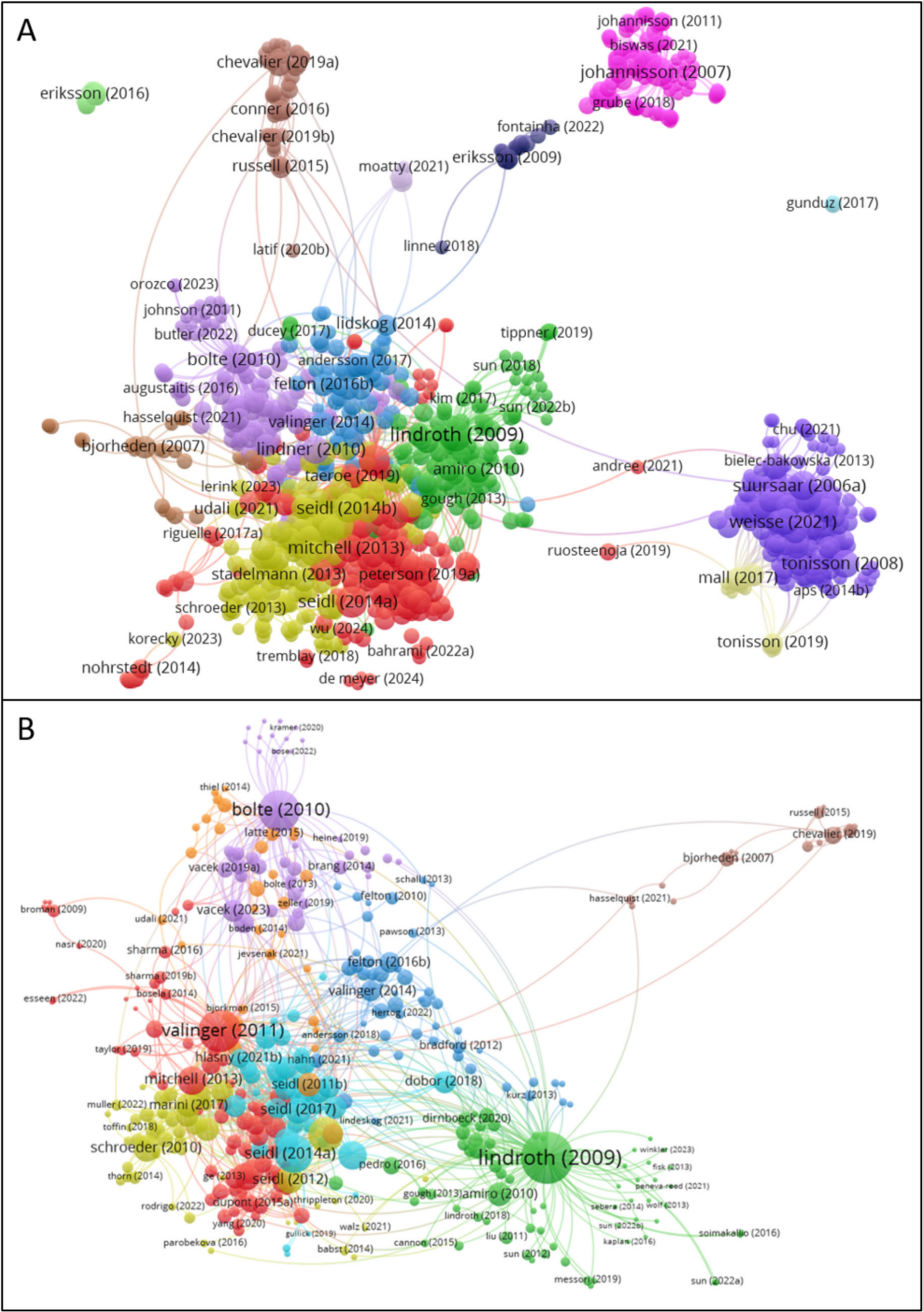
Network map of articles focusing on the storm Gudrun and citing articles. Panel “A” provides the result output from the general search, whereas panel “B” is focused on forest-related results

The initial clustering performed using the VOSviewer algorithm identified 16 distinct clusters, characterised by significant variation in citation patterns among them (Fig. 1A). Notably, research on storm Gudrun has extended beyond forest sciences, encompassing diverse domains such as oceanography, electoral studies, risk preparedness, and entrepreneurship (see SI for a full list of articles). Furthermore, studies conducted in countries along the eastern shores of the Baltic Sea, have emphasised different aspects of the storm’s impact, such as coastal storm surges. Despite this broad thematic diversity, the largest and most interconnected clusters predominantly comprise articles related to forests (Fig. 1B).

### Broadness within forest science research

The primary 18 forest-related research articles address a diverse range of topics. For instance, these articles addressed themes including wind dynamics (Seidl et al. 2014), impacts on forests and forest management (Valinger et al. 2019), storm effects on bird ecology (Russell et al. 2015), colonisation of storm-felled gaps by spruce bark beetles (Schroeder 2010), and timber logistics strategies to manage the crisis (Broman et al. 2009).

Our network map analysis revealed that numerous papers citing those primary 18 Gudrun articles largely followed the thematic trajectories established by the original works, resulting in eight clusters (Fig. 1B). This thematic differentiation was also notable in the most prevalent journals that cited these primary 18 Gudrun articles that were found within the eight clusters (Table 1). Interestingly, we noted under our characterisation exercise that authors in different clusters tend to treat the effects of storms either as disturbances or as damages, perhaps indicating a dichotomy between the ecological and silvicultural perspectives of storms (see label use in Table 1). The largest cluster “Wind, storms and forest damage” touched upon the central themes of storm–forest dynamics, such as the factors affecting windthrow. The second largest cluster “Storm disturbances and carbon dynamics” focused on the effects that storms have on the carbon cycle, and, in many cases, the broader implications for the European forest carbon sink. Another highly relevant cluster was “Spruce bark beetle and wind damage on wood /entomology”, which examined how damage to trees can be amplified when an abiotic disturbance (wind) increases the susceptibility of trees to subsequent biotic damages from bark beetle outbreaks. This cluster provided a clear bridge between the field of forest science and entomology. The most distinct cluster was characterised by a set of applied ecological studies that used Before and After Control methodologies to evaluate the implications of the storm on forest bird communities. This cluster also included papers on conservation ecology, riparian buffer ecology, and the bioeconomy.

**Table 1:** Clusters of forest science research articles derived from a citations network of storm Gudrun-related articles

Cluster (colour in Fig. 1B)	# Articles	Three most used journals	# Articles
1. Wind, storms, and forest damage (red)	75	Forest Ecology and Management	10
		Forests	10
		Forestry	8
2. Storm disturbances and carbon dynamics (green)	65	Global Change Biology	7
		Forest Ecology and Management	6
		Agricultural and Forest Meteorology	6
3. Forest management and adaptation to climate change—Sweden focus (dark blue)	52	Forest Ecology and Management	7
		Scandinavian Journal of Forest Research	5
		Forests	5
4. Spruce bark beetle and wind damage on wood/entomology (yellow)	50	Forest Ecology and Management	18
		Agricultural and Forest Entomology	3
		Plos One	3
5. Silviculture and storm effects, beech, spruce, mixed forest, Europe Focus I (purple)	42	Forest Ecology and Management	10
		Central European Forestry Journal	5
		European Journal of Forest Research	4
6. Ecology approach: storms as forest disturbances (light blue)	40	Forest Ecology and Management	7
		Global Change Biology	4
		Landscape Ecology	3
7. Disturbance effects and droughts, beech, spruce, Europe focus II (orange)	31	Forest Ecology and Management	5
		Forests	5
		Dendrochronologia	3
8. Applied ecology and conservation biology, and energy bioeconomy (brown)	25	Ecological Applications	3
		Biomass and Bioenergy	3
		Plos One	2

We found that at least three clusters could be further distinguished by differences in their geographical focus (Table 1). One cluster labelled “Forestry adaptation to climate change in southern Sweden”, included the large number of studies that were conducted specific to the regions impacted by Gudrun, and included research regarding its policy and adaptation implications. The other two clusters in which the geographical area was relevant draw attention to central Europe: “Silviculture and storm effects, beech, spruce, mixed forest, Europe focus I” and “Disturbance effects and droughts, beech, spruce, Europe focus II”—where the clusters’ labelling I and II stemmed from their distinct authorship and the degree to which drought was a central topic of concern.

In short, the thematical and geographical extent of the clusters illustrated how a phenomenon as specific and important as the storm Gudrun could be linked to a large number of research areas and in the past 20 years alone, to hundreds of studies citing the primary papers found through our search. It is also interesting how journal choice, geographical focus, scientific community grouping, and forms of describing the effects of storms resulted in such cluster diversity.

### Methodologies, funding, and environmental monitoring implications

The majority of the primary forest-related articles used quantitative rather than qualitative methods (16 articles). Moreover, we found that eight research articles were based on direct data collection, such as experiments and measurements in the field as well as interviews and document analysis, whereas the other articles used modelling or mixed research designs. Yet, more importantly, we found that 61% of the articles also relied on data from major environmental monitoring efforts. These include the national forest inventory (SLU), annual harvesting data, meteorological data (SMHI), the national Swedish bird survey that is part of the environmental monitoring programme of the Swedish Environmental Protection Agency and the county administrative boards, as well as, satellite images collected by the European Space Agency. For our analysis, we considered that any paper that relied upon data collected prior to the storm, could not, or would be very difficult (logistically and financially) to be recreated after the storm. We do, however, acknowledge that with enough financing and effort, one could in some cases develop proxies for data from before the storm. In other cases, for which the published articles did not depend explicitly upon such “before” data, it is reasonable to assume these studies could have still been conducted, although it is possible they were still indirectly reliant on other “beforehand” data collection that we did not identify. As such, the availability of a priori systematically collected data and networks was a central component of these studies and greatly enhanced the capacity of scientists to draw valuable insights from otherwise catastrophic disturbance events.

We found that research funding for the primary forest-related articles has mainly come from government agencies such as the Swedish Energy Agency, the Swedish Forest Agency, and the Swedish Environmental Protection Agency and from government research councils and foundations (Formas, VR, MISTRA). Other funding sources were from private foundations, EU research funds, or other EU states. Funding sources were not mentioned in five articles.

Overall, our findings highlight that an established infrastructure was instrumental in enabling researchers to study and learn from the catastrophic consequences of storm Gudrun. Pre-existing environmental monitoring systems, including systematic forest inventories and meteorological stations, provided critical baseline data. Additionally, targeted funding and a collaborative approach among stakeholders, characterised by a willingness to share experiences and data freely, created a conducive environment for advancing research on the storm’s impacts. This synergy between infrastructure, funding, and stakeholder openness facilitated the generation of important insights from such extreme events.

## Conclusion

Storm Gudrun has to a great extent influenced scientific research, particularly within those scientific fields that work with forests. Our analysis highlights how a singular catastrophic event like Gudrun can catalyse investigations across diverse research domains. We found that the initial wave of research was facilitated by robust pre-existing data sources and financial support from Swedish foundations and the European Union, underscoring the critical role of comprehensive environmental monitoring infrastructure and collaboration from the actors in the Swedish forest sector. Also, the availability of high-quality environmental data not only benefits research in Sweden, but also supported scientific communities throughout Europe.

The impact of abiotic and biotic disturbance events on Europe’s forests has increased over recent decades (Patacca et al. 2023) with substantial concerns regarding the additional risks and uncertainties posed to global forest systems from projected anthropogenic changes to the Earth’s climate (McDowell et al. 2020). It is, therefore, imperative to prioritise research that addresses these phenomena. This includes allocating funding specifically for the study of sudden, unpredictable events through mechanisms such as emergency grants. Equally important is societal recognition of the value of such research, reflected in continued investment in modern research frameworks and advanced environmental monitoring systems.

By prioritising and sustaining these efforts, we can expand our knowledge base, improve preparedness for future challenges, and extract valuable insights from adverse events. We hope that this communication demonstrates how important environmental monitoring is for building a solid scientific base that extends over national borders and thematic fields.

## Supplementary Information

Below is the link to the electronic supplementary material.

Supplementary file1 (XLS 6094 KB)

## Footnotes

1 https://www.vosviewer.com/.
